# Effect of Breed Purity and Rearing Systems on the Stability of Sliced Iberian Dry-Cured Ham Stored in Modified Atmosphere and Vacuum Packaging

**DOI:** 10.3390/foods10040730

**Published:** 2021-03-30

**Authors:** Rosario Ramírez, Rebeca Contador, Alberto Ortiz, Susana García-Torres, María Montaña López-Parra, David Tejerina

**Affiliations:** Meat Quality Area, Center for Scientific and Technological Research of Extremadura (CICYTEX-La Orden), Junta de Extremadura, Guadajira, 06187 Badajoz, Spain; mariarosario.ramirez@juntaex.es (R.R.); rebecontro@gmail.com (R.C.); alberto.ortiz@juntaex.es (A.O.); susana.garciat@juntaex.es (S.G.-T.); montana.lopez@juntaex.es (M.M.L.-P.)

**Keywords:** pre-packaged sliced Iberian dry-cured ham, commercial categories, pig breed, *Montanera*, shelf life, sensory evaluation

## Abstract

The long-term storage stability of three quality categories of sliced Iberian dry-cured ham defined by the Spanish Iberian Quality Standard (*Black*, purebred Iberian reared outdoors in Montanera system; *Red*, Iberian × Duroc crossed (50%) pigs reared outdoors in Montanera system; and *White*, Iberian × Duroc crossed (50%) pigs commercially fed) and packaged under vacuum and modified atmosphere packaging (MAP) was studied. Commercial category affected the shelf life, being *Black* and *Red* presented the highest lipid oxidation during storage, whereas the effect of packaging was not as clear as the effect of commercial category. MAP preserved more the colour and the antioxidants content than vacuum packaging, while this latter reduced lipid oxidation development and maintained better the brightness and flavour of slices than MAP.

## 1. Introduction

Iberian dry-cured products are highly appreciated not only by Spanish consumers [1], among whom they represent a relevant part in their diet, but also in the European market [2]. Iberian pigs have been traditionally reared outdoors in Mediterranean evergreen forests with feeding based on acorns mainly from *Quercus rotundifolia* and grass, namely the Montanera system [3], which results in the top quality Iberian products. Due to the growing demand for Iberian products, the Iberian sector has further diversified, including crossbreeding with Duroc breed [4] and the use of concentrate fed during the last fattening phase of the traditional Montanera system. Many research studies evaluated the impact of these production systems on the final product from a quality viewpoint, reporting the impact of the genetic background on fatty acid profile and sensory attributes [5] as well as on the physico-chemical characteristics and oxidative stability [6] of Iberian dry-cured ham. On the other hand, the feeding background of pigs affects the intramuscular fat, fatty acid and tocopherol profile of dry-cured hams [2,7]. This variety in the productive systems give rise to different quality standards in the final product, which are compiled in the current Spanish Iberian Quality Standard (IQS) [8] that regulates the Iberian breed purity and the rearing system, among other factors. This regulation classifies Iberian dry-cured products into four quality categories, which are commercially labelled with different colours: “*Black*” (100% Iberian pigs finished in Montanera), “*Red*” (a minimum of 50% Iberian pigs finished in Montanera), “*Green*” (a minimum of 50% Iberian pigs in outdoor system and fed with commercial fed and/or acorns and grass) and “*White*” (a minimum of 50% Iberian pigs in indoor systems and commercial fed). This could influence the technological aptitude of Iberian dry-cured hams. Consequently, it is important to know the effect of the commercial category in the shelf life of the sliced and packaged products.

The importance of new commercial formats of Iberian dry-cured ham has increased in recent years [1,9]. The traditional selling format as the whole piece implies that consumers should buy 5–7 kg of this product at once. This fact, together its high price, makes it difficult to purchase and consume, thus pre-packaged sliced Iberian dry-cured ham—especially vacuum and modified atmosphere packaging (MAP) [10]—has become popular. Specifically, for the Iberian dry-cured ham market, the selling format tends towards MAP format, since the product presentation is more similar to the original hand-sliced dry-cured ham and it reduces the slice adherence of the vacuum-packaging [10,11,12].

Although new selling formats provide more consumption flexibility and selling potential, the shelf life could decrease, since oxidative processes and alterations in colour and other nutrients are enhanced when the product is presented in sliced form, especially in MAP, as concluded Parra et al. [12]. In addition, Iberian dry-cured ham could present different shelf life according to its commercial category since the feeding background affects the antioxidants in the muscle [3]. Previous studies in dry-cured loin and chorizo have shown the importance of the commercial category of Iberian dry-cured meat products and the packaging conditions for the shelf life of the sliced product during storage [13,14].

Therefore, the aim of this research was to evaluate the differences in the main quality characteristics of pre-packaged sliced Iberian dry-cured hams from three commercial categories defined by the IQS. In parallel, we studied the shelf life of each commercial category and characterised the effect of the type of packaging—vacuum and MAP—through long-term refrigerated storage and retail display conditions.

## 2. Material and Methods

### 2.1. Material

#### 2.1.1. Iberian Dry-Cured Hams

Iberian dry-cured hams with three different commercial categories (*Black*, *Red* and *White*) defined by the IQS [8] were studied. According to these guidelines, pigs from *Black* and *Red* categories were reared under free-range system in dehesa ecosystem, during 70–80 days, with a replacement of 50–60 kg from the ad libitum intake of acorns and grass. In both, the age at slaughter was 15–16 months. The main differences between them were the Iberian breed purity (100% Iberian vs. 50% Iberian × Duroc) and the carcass weight (110–115 kg vs. 120–122 kg) for *Black* and *Red* category, respectively. Animals from *White* category (50% Iberian × Duroc) were reared under semi-intensive conditions, with a minimum total free floor area of 2 m^2^/animal during last fattening phase (70–80 days) and commercial feeding. The age at slaughter was 11–12 months with the carcass weight of 115–118 kg.

Raw hams were initially salted, and then progressively dried following the IQS [8] in a local facility, with 9.6 ± 0.6, 10.1 ±0.4 and 10.6 ± 0.7 kg of ham weight and 42, 42 and 30 months of maturation for *Black*, *Red* and *White*, respectively.

#### 2.1.2. Experimental Design

In total, 18 Iberian dry-cured hams from the three categories were used: *Black* (n = 6), *Red* (n = 6) and *White* (n = 6). Hams were hand-sliced at 1.5–3 mm thickness and homogeneously distributed in 100 g package formats.

Packaging of hams was carried out in a local facility. In total, 720 packages were evaluated (480 and 240 for physico-chemical and sensory analysis, respectively), 120 packages of each quality commercial category which were divided homogeneously to obtain the following groups: (i) vacuum-packaged sliced (n = 120 (80 and 40 for physico-chemical and sensory analysis, respectively)); and (ii) MAP (n = 120 (80 and 40 for physico-chemical and sensory analysis, respectively)). Packages of the three commercial qualities in both packaging conditions (vacuum packaging and MAP) were stored in refrigerated conditions (4 °C) for 12 months and studied every 4 months of storage: T0 (initial, n = 40 (20 + 20)), T4 (4 months of storage, n = 20 (20 + 0)), T8 (8 months of storage n = 40 (20 + 20)) and T12 (12 months of storage, n = 20 (20 + 0)).

#### 2.1.3. Packaging

Slices of dry-cured ham were vacuum-packaged using a laminated film (oxygen permeability, 9.3 cm^3^ O_2_/m^2^/24 h at 4 °C), using an EGARVAC^®^ packaging unit. MAP was carried out in a commercial mixture of gases with the proportion 70% N_2_-30% CO_2_ in an Ulma^®^ SMART 300 packaging equipment (Gipuzkoa, Spain), using polystyrene trays (150 mm thick) with an oxygen permeability of 3.2 cm^3^/m^2^/24 h at 4 °C and sealed with 70 mm thick polyethylene film (VIDUCA, Alicante, Spain) with an oxygen permeability of 1 cm^3^/m^2^/24 h (23 °C; 50% RH), 5.5 cm^3^/m^2^/24 h (23 °C; RH) to CO_2_ and 2.2 g/m^2^/24 h (25 °C; 90% RH) to H_2_O.

#### 2.1.4. Storage

All packages were stored at +4 °C under darkness for 12 months except in the last 72 h before their analysis, which occurred under fluorescent white light (616 LUX, 60 W) to reproduce commercial conditions. Packages are normally stored in darkness by the ham companies that slice the hams, and only when the packages are presented in the supermarket are they under illumination. For physico-chemical analysis, the sample unit was the packaging, so these were opened after the time of storage and the total content of each tray was homogenised, with an IKA homogeniser, except for the instrumental colour (which was measured on the intact slices) for subsequent analysis.

### 2.2. Methods

#### 2.2.1. Moisture and Chloride Content

Moisture was tested following the AOAC method [15] and chloride content (NaCl) using the Volhard method [16]. The results were expressed in g/100 g^−1^ of dry-cured ham as mean values.

Initial levels of fat content of packages were analysed and quantified gravimetrically with chloroform/methanol (2:1, *v*/*v*), following the method of Folch, Lees and Sloane-Stanley [17]. In total, 18 packages per commercial category were analysed. Total fat content of packages of hams of category were 18.0 ± 6.0 (*Black*), 17.4 ± 4.4 (*Red*) and 16.0 ± 6.0 (*White*) g 100^−1^. Those high levels of fat correspond to the sum of subcutaneous, intramuscular and intermuscular fat content. All these types of fat are present in each ham slice.

#### 2.2.2. Instrumental Colour Measurement

The parameters obtained in the CIE Lab space were lightness (L*), redness (a*, which evaluates the range of red to green) and yellowness (b*, which evaluates the range of yellow to blue) using a Minolta CR-400 colourimeter (Minolta Camera, Osaka, Japan) with illuminant D65, a 0° standard observer and a 2.5 cm port/viewing area. The equipment was standardised before developing the measurements with a white tile. Additionally, the saturation index or chroma (C*), defined as C = (a^2^ + b^2^)^0.5^, and hue angle (H°), as arctangent b*/a*, were calculated. The measurements were repeated at five randomly chosen places on each package and averaged.

#### 2.2.3. Tocopherols Composition

Tocopherols (α and γ) were quantified following the method described by Cayuela, Garrido, Sancho Bañón and Ros [18]. Extraction was carried out by saponifying solution (KOH 11.5% in EtOH/H_2_O 55:45). Tocopherol analysis was performed on an Agilent Technologies HPLC Series 1100 instrument (Agilent Technologies, Santa Clara, CA, USA), with a Kromasil Silica column (5 µm particle size, 150 × 4.6 cm) (Symta, Madrid, Spain) and a Kromasil Silica Guard Column (10 µm) (Symta, Madrid, Spain). The mobile phase was hexane:isopropanol:etanol (98.5:1:0.5 *v/v/v*), at a flow rate of 1 mL/min, and the fluorescence detector (Agilent Technologies Series 1200) was fixed at λ-excitation of 295 nm and λ-emission of 330 nm. Identification and quantification of the tocopherol compounds were made by comparison with standards analysed in similar conditions (0.2–14 µg/mL). The results were expressed as µg g^−1^.

#### 2.2.4. Lipid Oxidation

Lipid oxidation was evaluated by the 2-thiobarbituric acid (TBA) method of Salih, Smith, Price and Dawson [19]. TBARS values were determined from the standard (1,1,1,3-tetraethoxypropane, TEP) curve and expressed as mg malondialdehyde (MDA) kg^−1^.

#### 2.2.5. Protein Oxidation

Protein oxidation was evaluated following the method described by Oliver, Ahn, Moerman, Goldstein and Satadtman [20]. The formation of carbonyl groups during incubation with 2,4-dinitrophenylhydrazine (DNPH) in 2 N HCl was analysed. Carbonyls levels were determined by measuring DNPH incorporated on the basis of absorption of 21.0 mM^−1^ cm^−1^ at 370 nm for protein hydrazones. The results were expressed as nmol of DNPH fixed per mg of protein. Protein concentration was calculated by spectrophotometry at 280 nm using bovine serum albumin (BSA) as standard. Protein oxidation was expressed as nmol carbonyls mg protein^−1^.

#### 2.2.6. Fatty Acids Profile

The fatty acids composition was analysed in the fat previously extracted following the method of Folch et al. [17]. From this, 210 µL were taken and mixed with 4 mL of hexane and 200 µL of KOH (85% in MetOH). It was mixed and centrifuged (at 3000 rpm for 10 min), and, then, the organic phase was collected in vials. One microlitre was injected into a gas chromatograph equipped (model 4890 Series II; Hewlett-Packard, Palo Alto, CA, USA) with a split/split-less injector and a flame ionisation detector. A Carbowax™ fused silica capillary column (30 m × 0.25 mm id; 0.25 μm film thickness; Ohio Valley, Marietta, OH, USA) was used for the separation of FAMEs. The carrier gas was nitrogen at 1.8 mL min^−1^. The oven temperature was held at 200 °C. The injector and detector were set at 250 °C. The identification of individual FAME was based on a standard mixture of 37 Component FAME Mix (Sigma-Aldrich, Supelco 37 Component FAME Mix- CRM47885, St. Louis, MO, USA). Results were expressed as percentage of FAMEs in dry-cured ham sample, as mean values.

#### 2.2.7. Sensory Analysis

Prior to sensory analysis, microbiological analysis was carried out to confirm the microbial safety of slices before testing. To ensure microbial safety, total mesophiles, coliforms, *E. coli*, *Clostridium perfringens* and *Staphylococcus aureus* counts, as well as presence/absence of *Listeria monocytogenes* and *Salmonella* sp., were carried out on 5 random packages before sensorial analysis. Microbiological counts determined that the samples at the time of sensory analysis were suitable for consumption. Eight trained panellists evaluated nine sensory parameters of Iberian dry-cured ham slices. From each package, two slices were provided to each panellist as a representative as possible sample, being of sufficient size, similar intramuscular and subcutaneous fat and without visual defects. Sensory analysis was carried out at the beginning (T0) and after 8 months of refrigerated storage (T8). The visual appearance was assessed by means of two parameters: brightness (from little bright to intense) and marbling (level of visible intramuscular fat: from very lean to very marbled). The intensity of the typical flavour of dry-cured ham was also evaluated (from odourless to intense odour). The texture perceived while chewing was assessed according to hardness (from very tender to very firm) and juiciness (from not juicy to very juicy). Other parameters evaluated were the intensity of tastes (from tasteless to tasteful) such as salty, rancidity, unpleasant or strange tastes and the persistency of the typical flavour of dry-cured ham in mouth (from not perceptible to very persistent). In each session, samples of vacuum and MAP from the three commercial categories were evaluated. Samples were randomly presented to panellists. All sessions were conducted at ambient temperature in a sensory room equipped with white fluorescent lighting. Panellists evaluated the different parameters by means of a quantitative-descriptive analysis on a scale from 0 to 10. Water (approximately 100 mL) at room temperature was provided to the panellists between samples.

#### 2.2.8. Statistical Analysis

**Figure 1:**
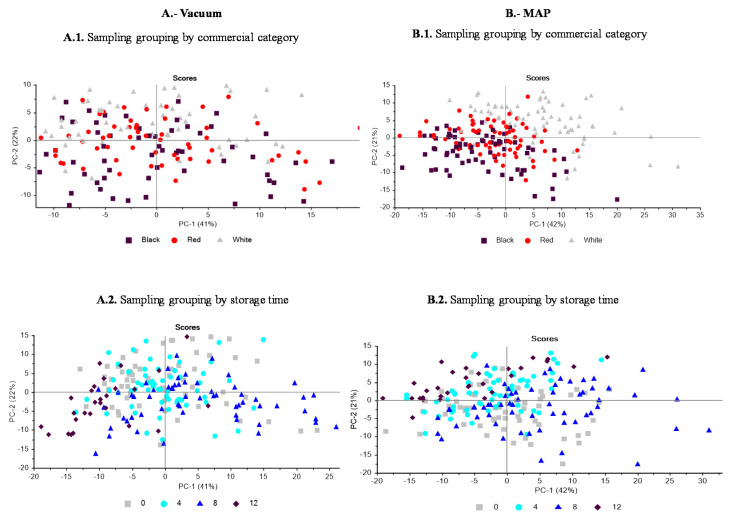
Principal component analyses (PCA) of Iberian dry-cured ham from three commercial categories (*Black*, *Red* and *White*) and different refrigerated (4 °C) storage time (T0, T4, T8 and T12) packed under: vacuum (A); and modified atmosphere (MAP) (70% O_2_:30% CO_2_). (**A**). PCA for vacuum packaging, using α- and γ-tocopherols, (−) C16:0, (−) C18:0 and C18:1 n-9 (PC1) and L*, C18:2 n-6 and C18:3 n-3 (PC2). (**B**). PCA of MAP, using (−) α and (−) γ-tocopherols, C16:0, C18:0 and (−) C18:1 n-9 (PC1) and (−) C18:2 n-6 and (−) C18:3 n-3 (PC2). Sampling grouping by commercial category is shown in (**A.1**,**B.1**): *Black*, *Red* and *White* are presented as black, red and grey markers, respectively. It is shown by storage time in (**A.2**,**B.2**): T0, T4, T8 and T12 are presented as grey, light blue, dark blue and black markers, respectively.

**Figure 2:**
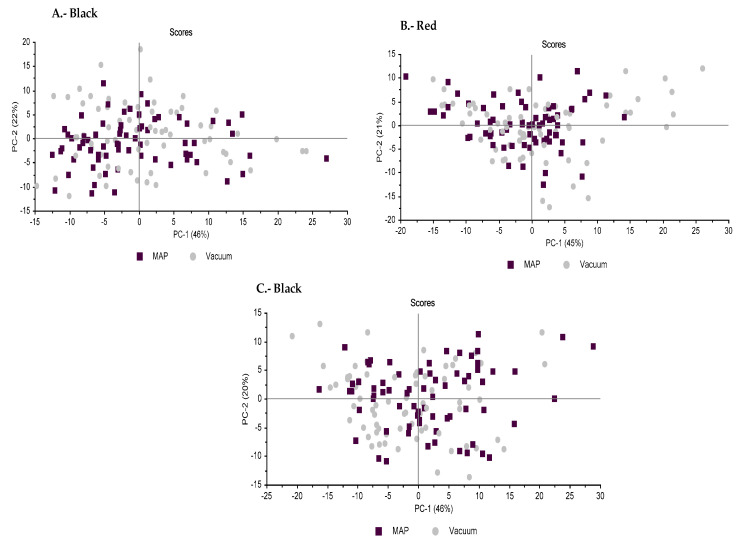
Principal component analyses (PCA) of Iberian dry-cured ham from two packaging format (vacuum and MAP) of three commercial categories: *Black* (**A**); *Red* (**B**); and *White* (**C**). Sampling grouping by type of packaging: Vacuum (grey markers) and MAP (black markers). (**A**) PCA of *Black* category, using (−) C16:0, (−) C18:0, C18:1 n-9 and C18:2 n-6 (PC1) and a*, Chroma, (−) lipid oxidation and (−) C18:3 n-3 (PC2). (**B**) PCA of *Red* category, using (−) L*, C16:0, C18:0 and (−) C18:1 n-9 (PC1) and (−) lipid oxidation, C18:2 n-6 and C18:3 n-3 (PC2). (**C**) PCA of *White* category, using C16:0, (−) C16:1, C18:0 and (−) C18:1 n-9 (PC1) and L*, b*, Hue and C18:2 n-6 (PC2).

A multivariate analysis of variance (Two-way ANOVA) was applied to data obtained using the software SPSS.PC+ v.20.0, taking into account the commercial category (*Black*, *Red* and *White*) and the type of packaging (vacuum vs. MAP) effects, and their interaction. One-way ANOVA test was also applied to analyse the effect of the storage at the beginning (T0), 4 months (T4), 8 months (T8) and 12 months (T12). In the sensory analysis, one-way ANOVA was applied to evaluate the effect of the storage (T0 and T8). Mean and standard error of mean (SEM) are reported. SEM include data of the commercial category and the type of packaging. Tukey’s HSD test was applied to compare the mean values of each group. Statistical significance was set at *p* ≤ 0.05. In addition, a principal component analysis was carried out using Unscrambler X (CAMO^®^ Trondheim, Norway) to check the overall effect of the commercial categories (*Black*, *Red* and *White*) and refrigerated storage time (T0, T4, T8 and T12) in each type of packaging (A (vacuum); and B (MAP)) (Figure 1) and the effect of packaging in each commercial category (Figure 2) ((A) *Black*; (B) *Red*; and (C) *White*) and to explore the multivariate relationships among variables.

## 3. Results and Discussion

Moisture and salt content of sliced dry-cured ham (Table 1) showed important changes due to the commercial category, while the type of packaging slightly influenced the parameters evaluated. Significant interactions between the commercial category and the type of packaging were found in moisture content at T8 and NaCl at T4. These are difficult to explain since they only appeared at specific times of storage.

Moisture content was the highest in slices from the the *Black* category (35.8 g 100 g^−1^); slices from the *Red* (37.5 g 100 g^−1^) showed intermediate values; and the lowest values were found in the *White* one (39.2 g 100 g^−1^). Differences could be associated to differences in the ripening times (42 months in *Black* and *Red* vs. 30 months in *White* categories) or inter- and intramuscular fat content. In general, the ripening time of these top-quality hams is quite long, and, in some cases, it could take several (3–5) years, which allows a slow moisture loss and the slow development typical maturation changes.

Regarding the effect of the type of packaging, after 12 months of storage (T12), slices in MAP showed significantly higher dry-matter content than vacuum-packaging, probably explained by the higher loss of moisture in the former. In fact, Parra et al. [10] found higher moisture content in slices of Iberian dry-cured ham packaged in vacuum in comparison with MAP after 60 days of storage. Iberian ham producers usually recommend the consumption of sliced hams after no longer than 6–8 months, principally due to the loss of sensory quality of the sliced ham, but there are no studies to support this.

Salt content values were within the range expected for this type of product [7], and their levels were similar in all categories at the beginning of storage (ANOVA did not detect differences), although Tukey test found the highest values in *White*, followed by *Red* category and the lowest values in *Black* category. During the storage, values of the slices from *Black* category were lower than those from the *Red* and *White* ones (*p* < 0.01), possibly associated to differences in the composition of the hams. The highest values of fat on slices were found in the *Black* category (18.0 ± 6.0 (*Black*), 17.4 ± 4.4 (*Red*), 16.0 ± 6.0 (*White*) g 100^−1^ sample), although differences were not significant (*p* > 0.05), which could have limited the salt penetration into the ham during salting process [21]. An interaction effect between both factors, commercial category and packaging, was observed at T4.

In general, instrumental colour showed important changes especially due to the commercial category, while the type of packaging slightly influenced the colour stability during storage (Table 2).

Lightness (L*) was mainly affected by the commercial category of ham but not by packaging. Significant interactions between commercial category and the type of packaging were found for L* at T8. Slices from the *White* category showed higher lightness than those from the *Black* one, with those from the *Red* category showing intermediate values. This pattern was maintained throughout the whole storage (T0–T12) and could result from the differences in meat composition (Table 1).

After 12 months of storage, L* decreased with regard of commercial category and packaging, although differences did not become significant for slices from the *Black* category. Reductions of lightness at the end of storage were less intense in slices from the *Black* category than from the others (ΔT12–T0: *Black* = 2.8; *Red* = 4.6; *White* = 3.6). In addition, reductions of lightness were less intense in MAP than in vacuum packaging (ΔT12–T0). In contrast, Parra et al. [10] did not find influence of packaging on lightness, although they reported a decrease of this parameter during storage due to illumination. Changes of lightness in the sliced dry-cured ham could be negative since modifications in the typical colour of dry-cured ham could influence consumers’ choice in the supermarket [22]. Therefore, the lower reduction of CIE L* in *Black* category is positive to preserve their original quality.

Redness (a*) at T0 was similar in all groups; however, at longer times of storage (T4–T12), it was significantly affected by the commercial category. Redness was higher in slices from the *White* and *Red* categories than from the *Black* one. Differences could be associated to differences in the ham composition and/or to the longer maturation times of hams from *Black* category. After storage, important reductions of a* were found with regard of the commercial categories, being higher in slices from the *Black* one (ΔT12-T0: *Black* = 3.3; *Red* = 2.8; *White* = 1.9). Long storage of dry-cured products can favour oxidation of the red pigments such as nytrosilmyoglobin to form metmyoglobin, leading to a progressive discolouration [23]. The decrease in red colour intensity could be negative for hams in the *Black* and *Red* categories as consumers prefer dry-cured hams with an intense red colour. Regarding to the effect of packaging, at T4, slices in vacuum packaging showed lower values of a* than those in MAP. In addition, at the end of storage, the reductions of a* were higher in vacuum packaging than in MAP (ΔT12-T0: MAP = 1.3; Vacuum = 3.0), thus expressing the relevance of the packaging characteristics and especially on the permeability to oxygen on the stability of the red colour of ham [24].

Changes in the red colour of dry-cured ham are normally attributed to the oxidation of nitrosylmyoglobin [23], since this molecule is unstable in the presence of oxygen [25]. Thus, when nitrosylmyoglobin is oxidised, the metmyoglobin is formed and redness turns to a brownish colour [23].

Yellowness (b*) was affected by the commercial category but not by packaging, leading to higher values in slices in *White* than those in *Black* category, while the *Red* ones presented intermediate values. During storage, b* decreased in all commercial categories and packaging. Decreases of yellowness are difficult to explain since normally the bibliography associates the increases of b* in sliced Iberian ham to increases of lipid oxidation during storage [10]. However, in line with our results, Amaro-Blanco, Delgado-Adámez and Ramírez [26] also found reductions of b* in sliced Iberian dry-cured shoulder (with similar characteristics to *Black* category) throughout storage time, although differences were not significant due to the shorter time of storage (five months).

Chroma and hue showed changes due to the modifications of the previous colour parameters. At T4–T12, chroma was significantly higher in slices from the *Red* and *White* categories than from the *Black* one. Chroma was initially higher in vacuum-packaged slices than in MAP at T0, while, at the end of storage (T12), it showed the opposite behaviour. Finally, hue angle was importantly reduced during storage in all commercial categories and packaging. A significative interaction at T8 was observed for the latter between commercial category and packaging.

Instrumental colour was affected by the commercial category of hams, which involves the genotype and feeding/production conditions. In general, the effect of both feeding and rearing on muscle colour of the dry-cured ham is complex, since colour parameters are influenced by different factors such as the oxidation intensity, the meat composition (moisture, fat and heme pigment content) and nitrite concentration [25,26,27]. However, the literature describes specific changes in some colour parameters. L* values were lower in dry-cured Iberian hams from animals reared outdoor (*Black* and *Red* categories), which was also observed by Isabel Cordero, López-Bote and Daza [28].

Despite the fact that shelf life of Iberian dry-cured ham is relatively long (6–8 months), research studies concerning the evolution of colour attributes of either vacuum packing or MAP deal with the effect of mid-term storage, approximately 2–4 months [10,11,12]. Only Cilla, Matínez, Beltrán and Roncalés [29] evaluated dry-cured ham quality and acceptability under vacuum or MAP during eight months, but not from Iberian pigs and with a different gas mixture in MAP to the mixture used in the present study. In line with our results, Parra et al. [12] and Parra et al. [10] reported a great stability in the lean colour in Iberian dry-cured ham slices in MAP at similar conditions as in the current study (70% N_2_-30% CO_2_) for up to 120 days of storage. In that latter study, they also compared both types of packaging (vacuum vs. MAP), reporting that vacuum-packaging preserved colour of sliced Iberian dry-cured ham better than MAP for 60 days of storage. Amaro-Blanco et al. [26] reported that the instrumental colour of slices Iberian dry-cured shoulder remained unchanged after 150 days of storage using vacuum packaging.

The antioxidants contents (Table 3) of the slices of Iberian dry-cured ham were importantly influenced by the commercial category, which in turn is affected by the genotype and the diet. α-tocopherol content was significantly higher at all sampling times (T0–T12) in slices from the *Black* category than from the *White* one, while the *Red* category showed intermediate levels. Regarding packaging, MAP showed higher levels of α-tocopherol at T0, T4 and T12. γ-Tocopherol was significantly higher in slices from the *Black* and *Red* categories than those from the *White* one at all times of storage (T0–T12). However, levels were importantly reduced during storage in slices from the *Black* category. In addition, at T0, γ-tocopherol content was higher in MAP than in vacuum packages.

Differences in the antioxidant content due to commercial categories could be explained by the differences of feeding background of pigs, with acorns and grass increasing the content of antioxidants in muscle. More specifically, the α-tocopherol content in muscles from pigs reared in the Montanera system is associated with the richness in α-tocopherol of the grass, whereas γ-tocopherol is linked to the acorns [3]. Differences found in α-tocopherol between slices from the *Black* and *Red* categories may be associated to differences in α-tocopherol content in the grass that pigs consumed, as reported by Tejerina, García-Torres, Cabeza de Vaca, Vázquez and Cava [30]. The antioxidant content of meat has technological implications in its stability, since tocopherols (α- and γ-isomers) levels present in the muscle affect the susceptibility to lipid oxidation of different tissues in vivo, in vitro and post-mortem [31].

Lipid oxidation development (Table 3) was higher in slices from hams of the *Black* category than those from the *White* category, while those from *Red* category had intermediate values of TBARS throughout the whole storage period. These differences are unexpected since the bibliography has reported a higher oxidative stability of Iberian hams from pigs reared outdoors [3,7]. Probably the longer maturation time of hams from the *Black* than the *White* category could explain these differences since lipid oxidation increases along maturation [32]. In addition, at T4–T12, the type of packaging also affected the TBARS values, which were higher in MAP than in vacuum packages. Significant interactions between commercial category and the type of packaging were found at T0 and T8. These could indicate that the lipid oxidative pattern of the commercial categories were different in each type of packaging (vacuum vs. MAP). TBARS values increased in all groups during storage, but they were more marked at T8 and T12. The increase between the beginning and the end of storage, were similar in all categories but slightly higher in the *Red* one (ΔT12-T0: *Black* = 0.6; *Red* = 0.8; *White* = 0.5). The type of packaging caused a similar increase in the TBARS values after 12 months of storage (ΔT12-T0: MAP = 0.7; Vacuum = 0.6). Other authors have also reported an increase in TBARS values in Iberian sliced dry-cured ham in MAP and vacuum packaging during storage [10,12]. In fact, plastic envelopes in packages have some permeability to oxygen. Increases of TBARS were also reported by Amaro-Blanco et al. [26] during long storage of sliced Iberian dry-cured shoulder in vacuum packaging. The progressive increase of lipid oxidation during long times of storage could be caused by some oxygen permeability of packages and by illumination, which allows the development of oxidative reactions. Regarding the benefits of MAP or vacuum packaging for sliced Iberian dry-cured hams, Parra et al. [10,12] concluded that the preservation of lipid oxidative stability was best achieved using vacuum packaging rather than MAP (70% N_2_-30% CO_2_). Slices in MAP were also perceived as more rancid than vacuum packaged slices. Differences between those studies and ours would be caused by differences in the oxygen permeability, the illumination of slices and the differences in the characteristics of Iberian hams used to carry out the studies (i.e., rearing systems of pigs).

Similarly, to the trend showed for lipid oxidation, protein oxidation was significantly higher in hams from the *Black* category than those from the *Red* and *White* ones. In this case, the type of packaging utilised did not affect the increase of protein oxidation. Significant interactions between commercial category and the type of packaging were found at T8, in line with results of TBA-RS. At the end of storage, all groups increased protein oxidation, especially at T8 and T12. Generally, lipid and protein oxidation follow the same trend since both are affected by unsaturated fatty acids, pigments, transition metals and other compounds [33]. Cava, Ladero, González, Carrasco and Ramírez [34] reported that lipid and protein oxidation levels increased after 90 days of storage in vacuum sliced dry-cured ham from pigs fed with concentrate. However, Amaro-Blanco et al. [26] found that TBARS values increased in sliced Iberian dry-cured shoulder in vacuum-packaging from pigs reared in the Montanera system, whereas no increases in protein oxidation were found after 150 days of storage. Our results suggest that lipid oxidation reactions could be initiated before the protein oxidation reactions, which are observed after long times of storage, especially after eight months.

The fatty acids profile (Table 4) was significantly affected by the commercial category, and, as a result, it was affected by the breed and/or the diet. Slices from the *White* category had significantly highest percentages of total saturated fatty acids (SFA) such as palmitic and stearic acids. In contrast, hams from the *Black* and *Red* categories presented the highest percentages of total monounsaturated fatty acids (MUFA) as well as oleic acid and polyunsaturated fatty acids such as linolenic acid. The sum of polyunsaturated fatty acids (PUFA) was the highest at initial times (T0 and T4) in the *Black* and the *Red* hams, while the lowest percentages were found in the *White* hams. However, at longer times of storage than T4, these differences in the sum of PUFA did not appear. Interactions between factors of analysis (commercial category vs. type of packaging) were significant at T0 for some fatty acids such as C18:0 and C18:3 n-3; and, during storage, such as C16:1 (T8 and T12) and C18:1 n-9 (T4, T8 and T12). Interactions at T0 are difficult to explain; however, interactions during storage could be caused by differences in the lipolytic effect of enzymes in slices from each category due to the type of packaging, which could cause changes in the fatty acids’ percentage during storage.

Fatty acids profile is associated with the feed of pigs. The fatty acids profile of the dry-cured ham from the *Black* and *Red* categories is in line with that reported for free-reared pigs fed during the final fattening period in a variable expanse of land, using natural resources, grass and acorns [35]. The fatty acids profile of hams from the *White* category is dependent on the composition of the commercial fodder, which is accordance with that reported for hams from pigs with similar genetic [36]. Results of the fatty acids profiles agree with previous studies of Tejerina et al. [3], who evaluated the influence of the diet on the fatty acids profile of muscle of Iberian pigs reared in the Montanera system, which would correspond to the *Black* and *Red* categories, and commercial fodder, which would correspond to the *White* category.

Percentages of individual fatty acids and their ratios were significantly modified during storage: SFA tended to increase during storage, while MUFA and PUFA decreased. The ratio n-6/n-3 was importantly increased during storage, probably due to the important reduction of n-3 fatty acids such as linolenic acid, as a result of increased oxidation, since the oxidation susceptibility is correlated exponentially with the level of unsaturation of fatty acids, hence the oxidation rate is higher in n-3 than in n-6 polyunsaturated fatty acids [37].

The fatty acids profile also affects the oxidative stability of dry-cured hams since high levels of MUFA make them more stable during the maturation process. The balanced content of antioxidants and MUFA improves the technological quality of meat [38].

Other studies have reported increases of free fatty acids during storage in sliced vacuum packaged dry-cured ham [39]. The decreases of PUFA and linoleic acid during storage could be caused by their release as free fatty acids by the action of lipolytic enzymes. The higher ratio n-6/n-3 during storage and the higher decrease of PUFA and linoleic acid in dry-cured ham from pigs commercially fed (*White* category) makes these more sensitive to lipid oxidation reactions. PUFA, contrary to MUFA, are very easily oxidised, leading to the formation of compounds that favours the development of rancidity and undesirable sensory perceptions [38].

Lipolysis is one of the main pathways for the formation of the typical characteristics of dry-cured ham since the free fatty acids from lipolytic reactions are rapidly oxidised and new aromatic compounds are formed [40]. However, the compounds formed during storage of sliced dry-cured ham would negatively affect to the original aromatic profile of Iberian dry-cured ham.

In the sensory analysis (Table 5), the brightness was significantly affected at T8 by the type of packaging. Slices in vacuum packaging were brighter than those in MAP, and the brightness of the ham in those packages (vacuum) was increased during storage. Brightness is a parameter related to the composition of intramuscular fat. Fat of dry-cured ham from pigs reared in the Montanera system are expected to present high brightness due to a high level of MUFA and oleic acid; however, despite the differences showed in the fatty acids profile, panellists did not appreciate differences in brightness. The reduction of the brightness in slices of ham is negative for a packaged ham, so vacuum packaging would be better than MAP at least for this parameter.

Only the sensory parameter “intensity of marbling” was significantly affected by the commercial category. Hams in the *Black* category had lower marbling than those in the *White* category, while the *Red* category showed intermediate levels. Marbling is mostly associated with intramuscular fat content, and this parameter shows an important variability. Hams with *Black* category are expected to have higher marbling since Iberian pigs reared in the Montanera system usually have higher intramuscular fat content than those commercially feeding [41]. These results could also be associated to the generally higher life weight of pigs reared in the Montanera system with respect to the semi-intensive systems [42]. In addition, there is a great variability between the different genetic lines of Iberian pigs [43], which also affects the quality of dry-cured meat products [44], as well as the Duroc line [34,38] used in animals from the *Red* and *White* categories.

The intensity of flavour was also affected by the type of packaging and higher scores were found in vacuum packaging than in MAP at T8. In addition, this parameter decreased after eight months of storage in all commercial categories of ham. Iberian dry-cured ham is appreciated for its sensory properties; therefore, it is important to know the long-term evolution of these parameters, especially for the Iberian manufacturing sector and export market [45], as well as by consumers, since this ham is considered by them as a top-quality product. Eight months of storage is a long storage time for Iberian sliced dry-cured ham. The decrease of flavour perceived by panellists would be in line with the recommendations to consume sliced Iberian ham before 6–8 months, not due to microbiological problems, but because the sensory quality of the ham starts to decrease significantly after that time, e.g., the reductions of flavour intensity or the development of undesirable flavours. However, as far as we know, there are no studies on the sensory changes in sliced dry-cured ham stored longer than five months [26,46]. Lipid oxidation is the principal route for the formation of volatile aromatic compounds in Iberian dry-cured meat products during maturation; thus, to a certain level, lipid oxidation is positive [40], even though compounds produced during storage from lipid oxidation are considered negligible.

Texture parameters such as hardness and juiciness were not affected by the commercial category or the type of packaging.

Hams packaged in MAP were perceived as saltier than those in vacuum packaging. The results are difficult to explain since saltiness is mostly related to salt content. These changes could be influenced by the higher dehydration of hams in MAP than those in vacuum packaging. In general, salt perception was increased during storage although changes were only significant for the *Black* and *White* categories.

The intensity of rancidness perception was not affected by the commercial category or the type of packaging of sliced ham. However, during storage, the slices of *Red* and *White* commercial categories and the slices in MAP were perceived significantly more rancid after eight months of storage than at the beginning. Very rancid hams are considered defective. Ruiz, García, Muriel, Andrés and Ventanas [47] reported a certain negative influence of rancidity on the acceptability of Iberian ham. The increases of rancidness agree with the increases in TBARS values during storage; however, despite of their higher TBARS values at T12, slices from the *Black* category were more stable during storage than those from the other categories. In this line, slices of Iberian dry-cured ham from pigs reared outdoors with acorns and grass with high content of antioxidants [30] would be generally more stable since antioxidants are incorporated into muscle [48]. This type of ham could present a better technological aptitude for processes such as slicing, packaging and processing (i.e., high pressure treatment) than those hams from pigs with other rearing systems, as suggested Amaro-Blanco et al. [26] considering sliced Iberian dry-cured shoulder from pigs reared in the Montanera system that were vacuum packaged and treated by high pressure processing. Regarding the type of packaging, Cilla et al. [29] and Parra et al. [10] found that the flavour was better preserved in vacuum rather than MAP, which would agree with the significant increase of rancidness in MAP at T8 compared to T0, while vacuum packaging showed similar scores at both times. Amaro-Blanco et al. [26] found that sensory parameters of sliced Montanera Iberian dry-cured shoulder were not affected after five months of storage.

The perception of strange flavours was increased in all groups during storage, although differences were only significant for slices from the *Red* category. On the other hand, the persistency of the flavour in the top and bottom of the mouth is a positive attribute for high quality Iberian dry-cured hams. The increase of persistency during storage could be also linked to the increase of lipid oxidation-derived compounds.

The principal component analysis about the different commercial categories of Iberian dry-cured ham at different packaging conditions (Figure 1) revealed some differences in the sample distribution. In vacuum packaging, the discrimination was not as clear (Figure 1A.1) as in MAP, which allowed the separation of individuals according to their commercial category (Figure 1B.1). Thus, the last one showed how samples from *White* category tended to have positive scores on the principal component (PC) 1 and 2 axes, which could be explained by positive loadings of the variables C16:0, C18:0 and SFA on the PC1 axis. On the contrary, no discrimination was observed among the samples derived from the *Red* and *Black* categories, which provided samples located in the mid-zone of the map and even tended to have negative scores on both axes, as explained by the negative loadings of the variables α- and γ-tocopherol, C18:1 and MUFA (PC1) and C18:2, C18:3 and PUFA (PC2). These results support the stronger influence exerted by the production system—Montanera—compared to the purity breed on the composition of antioxidants and fatty acid profile in Iberian dry-cured hams. Figure 2A.2,B.2 shows the evolution of packages during storage. Vacuum packaging samples stored for 12 months were clearly located on the left of the PC1, which could mainly be explained because of the negative loadings of SFAs on the PC1 axis. MAP samples at 12 months of storage had positive scores on the PC2 axis, which could be due to decrease of PUFA during at the end of the storage time of the study.

When the packaging effect in each commercial category was explored, the projection of the samples onto the space defined by PC1 and PC2 (Figure 2) showed that the model did not discriminate between both packaging types—vacuum and MAP—regardless of the commercial category considered, *Black* (Figure 2A), *Red* (Figure 2B) or *White* (Figure 2C). Henceforth, the relative position of the samples suggests that physico-chemical differences accounting for type of packaging were not enough to allow the discrimination of the three commercial categories under study. The overall effect evaluated through the PCA analysis would confirm the higher influence of the commercial category (which is affected by the rearing system and the genotype) over the packaging conditions (vacuum vs. MAP) on the quality traits of sliced Iberian dry-cured ham.

## 4. Conclusions

Iberian dry-cured ham from different commercial categories, established on the basis of genetic and feeding/production conditions, presented important differences on antioxidants and fatty acids profile, being tocopherols and monounsaturated fatty acids higher in those from animals reared in the Montanera system. However, these slices did not better maintain the colour than those from pigs commercially fed during long storage times. During storage, the fatty acids profile showed important changes, probably due to the lipolytic reactions, which could be more intense in hams from pigs commercially fed. Despite the initial higher levels of protein and lipid oxidation in dry-cured ham slices from the *Black* category, they did not develop higher rancidness than those from the other categories.

MAP better preserved the colour and the antioxidants content than vacuum packaging, while vacuum packaging reduced lipid oxidation development and the brightness and flavour of slices. The improvement in the future of these packaging types (i.e., active packaging and packages permeability to oxygen) could increase the shelf life of sliced Iberian dry-cured ham.

## Figures and Tables

**Table 1:** Moisture content (g 100 g^−1^ of ham) and NaCl (g 100 g^−1^ of ham) of sliced Iberian dry-cured ham from different commercial categories and with different packaging conditions for 12 months of refrigeration storage.

		Commercial Category (1)			Packaging (2)		SEM	*p* Value		
		*Black*	*Red*	*White*	MAP	Vacuum		1	2	1 × 2
N total *		*80*	*80*	*80*	*120*	*120*				
**Moisture**	T0	35.8 a	37.5 b	39.2 c	37.5	37.5	0.29	0.001	0.967	0.587
	T4	35.9 a	38.3 b	39.8 c	37.5	38.5	0.29	0.001	0.065	0.191
	T8	34.8 a	38.0 b	39.6 c	37.5	37.5	0.31	0.001	0.923	0.019
	T12	35.7 a	38.3 b	39.6 c	36.6	39.1	0.43	0.001	0.001	0.704
	SEM	0.22	0.24	0.27	0.21	0.25				
	*p* value	0.180	0.565	0.901	0.465	0.066				
**NaCl**	T0	4.1 c	4.2 b	4.3 a	4.2	4.2	0.04	0.060	0.512	0.377
	T4	4.1 b	4.3 a	4.2 ab	4.3	4.2	0.03	0.007	0.149	0.007
	T8	4.1 b	4.3 a	4.3 a	4.2	4.2	0.04	0.003	0.708	0.144
	T12	4.1 b	4.3 a	4.3 a	4.2	4.2	0.05	0.009	0.889	0.872
	SEM	0.03	0.03	0.04	0.02	0.03				
	*p* value	0.956	0.600	0.711	0.946	0.861				

SEM, standard error of mean; Values with the same letters (a–c) indicate homogeneous subsets according to *p* ≤ 0.05, *p* ≤ 0.01, *p* ≤ 0.001. a–c: Different letters in the same row indicate significant differences in the commercial category for *p* = 0.05 according to Tukey’s HSD test. *Black* (100% Iberian pigs finished in Montanera); *Red* (50% Iberian × Duroc pigs finished in Montanera); *White* (50% Iberian × Duroc pigs reared in a semi-intensive system with commercially feeding). Modified atmosphere packaging (MAP) (70% N_2_:30% CO_2_). Vacuum packaging. T0, initial; T4, 4 months of storage; T8, 8 months of storage; T12, 12 months of storage. * N total, number of determinations performed for each commercial category and for each type of packaging.

**Table 2:** Instrumental colour changes of sliced Iberian dry-cured ham from different commercial categories and with different packaging conditions for 12 months of refrigeration storage.

		Commercial Category (1)			Packaging (2)		SEM	*p* Value		
		*Black*	*Red*	*White*	MAP	Vacuum		1	2	1 × 2
N total *		*80*	*80*	*80*	*120*	*120*				
L *	T0	39.9 b	41.8 abAB	43.9 aA	42.4 A	41.3 A	0.52	0.004	0.262	0.252
	T4	39.4 b	39.4 abB	43.7 aA	41.2 A	40.5 A	0.33	0.173	0.057	0.286
	T8	39.9 c	43.3 bA	44.3 aA	43.9 A	42.5 A	0.56	0.001	0.150	0.006
	T12	37.1 b	37.2 bC	40.4 aB	39.1 B	37.3 B	0.58	0.018	0.098	0.062
	SEM	0.42	0.40	0.50	0.37	0.37				
	*p* value	0.162	0.001	0.001	0.001	0.001				
a *	T0	22.4 A	22.9 A	23.1 AB	22.1 A	23.5 A	0.38	0.747	0.058	0.368
	T4	20.8 bA	23.4 aA	24.1 aA	23.2 A	22.3 A	0.26	0.001	0.041	0.619
	T8	18.4 bB	19.5 abB	20.8 aC	19.8 B	19.4 B	0.32	0.008	0.514	0.111
	T12	19.1 bB	20.1 aB	21.1 aC	20.8 B	20.5 B	0.40	0.005	0.128	0.546
	SEM	0.34	0.30	0.30	0.24	0.28				
	*p* value	0.001	0.001	0.001	0.001	0.001				
b *	T0	13.1 bA	14.1 abA	15.1 aA	13.6 A	14.6 A	0.27	0.011	0.077	0.409
	T4	12.6 bA	13.6 abA	14.7 aA	13.3 A	14.0 A	0.25	0.002	0.133	0.962
	T8	12.1 cA	13.8 bA	15.3 aA	13.7 A	13.8 A	0.26	0.001	0.802	0.077
	T12	9.3 bB	10.5 abB	12.6 aB	11.7 B	10.0 B	0.49	0.015	0.068	0.530
	SEM	0.23	0.25	0.28	0.21	0.23				
	*p* value	0.001	0.001	0.019	0.015	0.001				
**Chroma**	T0	26.1 A	27.0 A	27.7 AB	26.0 A	27.8 A	0.43	0.302	0.033	0.376
	T4	24.3 bAB	27.1 aA	28.6 aA	26.8 A	26.6 AB	0.33	0.001	0.732	0.678
	T8	22.2 bB	24.1 aB	25.7 aB	24.1 B	23.9 C	0.34	0.001	0.805	0.180
	T12	23.7 bAB	26.3 abA	28.4 aA	27.6 A	24.7 BC	0.54	0.001	0.004	0.815
	SEM	0.35	0.33	0.34	0.27	0.32				
	*p* value	0.001	0.002	0.008	0.001	0.001				
**Hue**	T0	30.7 A	31.8 B	33.2 B	31.7 B	32.0 B	0.46	0.102	0.751	0.526
	T4	31.2 A	29.6 B	31.2 B	29.3 B	32.1 B	0.42	0.177	0.001	0.749
	T8	33.6 A	35.1 bA	37.0 A	34.8 A	35.7 A	0.60	0.058	0.471	0.019
	T12	23.2 B	22.9 C	25.9 C	24.5 C	23.5 C	0.69	0.150	0.423	0.212
	SEM	0.53	0.54	0.56	0.53	0.46				
	*p* value	0.001	0.001	0.001	0.001	0.001				

SEM, standard error of mean; Values with the same letters (a–c or A–C) indicate homogeneous subsets according to commercial category and refrigerated storage time (T0, T4, T8 or T12) for *p* = 0.05 according to Tukey´s HSD test. *Black* (100% Iberian pigs finished in Montanera); *Red* (50% Iberian × Duroc pigs finished in Montanera); *White* (50% Iberian × Duroc pigs reared in a semi-intensive system with commercially feeding). Modified atmosphere packaging (MAP) (70% N_2_:30% CO_2_). Vacuum packaging. T0, initial; T4, 4 months of storage; T8, 8 months of storage; T12, 12 months of storage. * N total, number of determinations performed for each commercial category and for each type of packaging.

**Table 3:** Tocopherol content (µg g^−1^) and lipid (TBARS values) and protein oxidation changes of sliced Iberian dry-cured ham from different commercial categories and with different packaging conditions for 12 months of refrigeration storage.

		Commercial Category (1)			Packaging (2)		SEM	*p* Value		
		*Black*	*Red*	*White*	MAP	Vacuum		1	2	1 × 2
N total *		*80*	*80*	*80*	*120*	*120*				
**α-Tocopherol**	T0	11.2 a	8.7 b	4.0 c	8.8	7.1	0.37	0.001	0.001	0.060
	T4	10.5 a	7.9 b	4.0 c	7.8	7.1	0.31	0.001	0.049	0.338
	T8	10.5 a	8.0 b	4.0 c	7.8	7.2	0.31	0.001	0.111	0.510
	T12	9.9 a	8.1 b	3.7 c	7.7	6.8	0.39	0.001	0.008	0.502
	SEM	0.266	0.168	0.062	0.266	0.215				
	*p* value	0.477	0.359	0.473	0.426	0.833				
**γ-Tocopherol**	T0	1.1 aA	1.1 a	0.3 b	0.9	0.7	0.04	0.001	0.008	0.224
	T4	1.0 aAB	1.0 a	0.3 b	0.7	0.7	0.05	0.001	0.837	0.990
	T8	1.0 aAB	1.0 a	0.3 b	0.8	0.7	0.04	0.001	0.905	0.996
	T12	0.8 aB	1.0 a	0.3 b	0.7	0.7	0.05	0.001	0.787	0.981
	SEM	0.03	0.03	0.01	0.03	0.03				
	*p* value	0.035	0.724	0.744	0.279	0.836				
**mg MDA kg^−1^**	T0	1.9 aC	1.7 abC	1.6 bC	1.8 D	1.7 C	0.04	0.001	0.119	0.001
	T4	2.1 aC	1.8 abC	1.7 bBC	1.9 C	1.8 C	0.03	0.001	0.003	0.543
	T8	2.3 aB	2.1 abB	1.8 bB	2.2 B	2.0 B	0.03	0.001	0.001	0.001
	T12	2.5 aA	2.5 aA	2.1 bA	2.5 A	2.2 A	0.06	0.005	0.024	0.964
	SEM	0.03	0.04	0.03	0.03	0.03				
	*p* value	0.001	0.001	0.001	0.001	0.001				
**nmol carbonyls mg^−1^ protein**	T0	4.2 aB	3.7 bB	3.5 bB	3.8 B	3.7 B	0.04	0.001	0.119	0.227
	T4	4.2 aB	3.7 bB	3.5 bB	3.8 B	3.8 B	0.04	0.001	0.793	0.128
	T8	4.5 aA	3.8 bAB	3.8 bA	4.0 AB	4.1 A	0.05	0.001	0.324	0.019
	T12	4.5 aA	4.0 bA	3.9 bA	4.2 A	4.1 A	0.06	0.001	0.536	0.929
	SEM	0.04	0.03	0.03	0.03	0.03				
	*p* value	0.001	0.029	0.001	0.002	0.001				

SEM, standard error of mean; Values with the same letters (a–c or A–C) indicate homogeneous subsets according to commercial category and re-frigerated storage time (T0, T4, T8 or T12) for *p* = 0.05 according to Tukey’s HSD test. *Black* (100% Iberian pigs finished in Montanera); *Red* (50% Iberian × Duroc pigs finished in Montanera); *White* (50% Iberian × Duroc pigs reared in a semi-intensive system with commercially feeding). Modified atmosphere packaging (MAP) (70% N_2_:30% CO_2_). Vacuum packaging. T0, initial; T4, 4 months of storage; T8, 8 months of storage; T12, 12 months of storage. * N total, number of determinations performed for each commercial category and for each type of packaging.

**Table 4:** Fatty acids profile (per cent of FAMEs) changes of sliced Iberian dry-cured ham from different commercial categories and with different packaging conditions for 12 months of refrigeration storage.

		Commercial Category (1)			Packaging (2)		SEM	*p* Value		
		*Black*	*Red*	*White*	MAP	Vacuum		1	2	1 × 2
N total *		*80*	*80*	*80*	*120*	*120*				
**C16:0**	T0	22.0 bB	22.1 bB	23.9 aB	22.7 B	22.7 B	0.11	0.001	0.623	0.105
	T4	22.4 bB	21.9 cB	24.0 aB	22.5 B	23.0 B	0.12	0.001	0.075	0.108
	T8	22.2 bB	22.0 bB	23.3 aC	22.4 B	22.6 B	0.14	0.001	0.361	0.209
	T12	23.5 bA	22.9 cA	24.6 aA	23.4 A	23.9 A	0.14	0.001	0.087	0.150
	SEM	0.12	0.08	0.08	0.09	0.10				
	*p* value	0.005	0.002	0.001	0.007	0.001				
**C16:1**	T0	2.9 bAB	3.0 bA	3.7 aA	3.3 A	3.1 AB	0.05	0.001	0.064	0.158
	T4	3.0 bA	3.0 bA	3.8 aA	3.4 A	3.2 A	0.04	0.001	0.091	0.204
	T8	3.0 bA	3.0 bA	3.7 aA	3.2 A	3.3 A	0.05	0.001	0.654	0.002
	T12	2.7 bB	2.8 bB	3.3 aB	3.0 B	2.9 B	0.053	0.001	0.377	0.039
	SEM	0.03	0.03	0.04	0.04	0.03				
	*p* value	0.022	0.067	0.001	0.011	0.011				
**C18:0**	T0	10.1 bB	9.9 bB	10.9 aAB	10.3 B	10.3 B	0.09	0.001	0.786	0.012
	T4	10.8 bAB	10.4 bB	11.4 aAB	10.4 B	11.3 A	0.10	0.001	0.001	0.737
	T8	10.2 B	10.0 B	10.2 B	10.1 B	10.2 B	0.15	0.738	0.823	0.088
	T12	11.2 bA	10.9 bA	11.9 aA	11.1 A	11.6 A	0.11	0.001	0.082	0.916
	SEM	0.12	0.09	0.11	0.08	0.10				
	*p* value	0.014	0.001	0.001	0.001	0.001				
**C18:1 n-9**	T0	53.3 aA	53.8 a	50.5 b	52.6 B	52.5 AB	0.20	0.001	0.925	0.054
	T4	53.4 aA	53.8 a	50.7 b	53.2 A	52.0 B	0.22	0.001	0.001	0.939
	T8	53.4 aA	53.1 a	50.7 b	53.3 A	52.8 A	0.25	0.001	0.042	0.104
	T12	52.6 aB	53.5 a	50.6 b	52.8 B	51.7 C	0.24	0.001	0.002	0.614
	SEM	0.18	0.17	0.19	0.16	0.18				
	*p* value	0.016	0.064	0.081	0.028	0.001				
**C18:2 n-6**	T0	7.1 aA	6.6 ab	6.4 b	6.6	6.8	0.08	0.001	0.283	0.497
	T4	6.5 abB	6.9 a	6.3 b	6.6	6.5	0.08	0.002	0.484	0.249
	T8	6.7 AB	6.5	6.6	6.6	6.6	0.08	0.452	0.999	0.632
	T12	6.6 B	6.6	6.3	6.4	6.6	0.11	0.322	0.443	0.691
	SEM	0.08	0.09	0.05	0.06	0.07				
	*p* value	0.015	0.240	0.125	0.673	0.436				
**C18:3 n-3**	T0	1.1 aA	1.1 aA	0.8 bA	1.0 A	1.0 A	0.01	0.001	0.596	0.016
	T4	0.6 aB	0.6 aB	0.4 bB	0.5 B	0.6 B	0.01	0.001	0.065	0.062
	T8	0.5 aC	0.5 aC	0.3 bB	0.4 C	0.4 C	0.01	0.001	0.609	0.109
	T12	0.5 aC	0.5 aC	0.4 bB	0.4 C	0.5 C	0.017	0.001	0.278	0.244
	SEM	0.02	0.02	0.02	0.02	0.02				
	*p* value	0.001	0.001	0.001	0.001	0.001				
**SFA**	T0	34.2 bB	34.0 bB	36.8 aB	35.0 B	35.0 C	0.18	0.001	0.897	0.005
	T4	35.1 bAB	34.3 cB	37.4 aAB	35.0 B	36.2 B	0.20	0.001	0.001	0.461
	T8	34.5 bB	34.2 bB	35.9 aC	34.6 B	34.9 C	0.28	0.042	0.573	0.121
	T12	35.4 bA	35.3 bA	38.3 aA	36.3 A	37.4 A	0.23	0.001	0.004	0.457
	SEM	0.22	0.15	0.19	0.16	0.18				
	*p* value	0.005	0.001	0.001	0.001	0.001				
**MUFA**	T0	57.1 aA	57.6 aA	54.9 bA	56.7 B	56.5 B	0.19	0.001	0.525	0.023
	T4	57.2 aA	57.6 aA	55.4 bA	57.3 A	56.2 B	0.21	0.001	0.001	0.987
	T8	57.3 aA	57.2 aB	55.4 bA	57.5 A	57.2 A	0.26	0.025	0.442	0.047
	T12	56.8 aB	57.1 aB	54.4 bB	56.4 B	55.2 C	0.23	0.001	0.002	0.726
	SEM	0.19	0.17	0.21	0.15	0.18				
	*p* value	0.009	0.001	0.001	0.001	0.001				
**PUFA**	T0	8.7 aA	8.4 bA	7.9 cA	8.3 A	8.5 A	0.09	0.001	0.266	0.672
	T4	7.7 abB	8.1 aAB	7.4 bB	7.8 B	7.7 B	0.09	0.001	0.830	0.200
	T8	7.9 B	7.8 B	7.8 AB	7.8 B	7.8 B	0.09	0.237	0.957	0.548
	T12	7.5 B	7.7 B	7.3 B	7.4 B	7.5 B	0.12	0.144	0.411	0.652
	SEM	0.09	0.10	0.06	0.06	0.07				
	*p* value	0.001	0.010	0.001	0.001	0.001				
**n-6/n-3**	T0	7.2 bD	6.9 bC	8.4 aD	7.4 C	7.6 C	0.11	0.001	0.363	0.037
	T4	11.1 cC	12.6 bB	17.6 aC	15.0 B	12.5 B	0.32	0.001	0.001	0.001
	T8	14.3 cA	16.6 bA	22.3 aA	18.2 A	17.4 A	0.34	0.001	0.030	0.001
	T12	13.3 cB	15.9 bA	19.5 aB	16.4 AB	16.1 A	0.37	0.001	0.614	0.047
	SEM	0.23	0.36	0.46	0.35	0.30				
	*p* value	0.001	0.001	0.001	0.001	0.001				

SEM, standard error of mean; Values with the same letters (a–c or A–D) indicate homogeneous subsets according to commercial category and re-frigerated storage time (T0, T4, T8 or T12) for *p* = 0.05 according to Tukey´s HSD test. *Black* (100% Iberian pigs finished in Montanera); *Red* (50% Iberian × Duroc pigs finished in Montanera); *White* (50% Iberian × Duroc pigs reared in a semi-intensive system with commercially feeding). Modified atmosphere packaging (MAP) (70% N_2_:30% CO_2_). Vacuum packaging. T0, initial; T4, 4 months of storage; T8, 8 months of storage; T12, 12 months of storage. PUFA, polyunsaturated fatty acids (C18:2 n-6 + C18:3 n-3 + C20:4 n-6); MUFA, monounsaturated fatty acids (C16:1 + C17:1 + C18:1 + C20:1); SFA, saturated fatty acids (C12:0 + C14:0 + C16:0 + C17:0 + C18:0 + C20:0); n6 = C18:2 n-6 + C20:4 n-6; n3 = C18:3 n-3.* N total, number of determinations performed for each commercial category and for each type of packaging.

**Table 5:** Sensory analysis changes of sliced Iberian dry-cured ham from different commercial categories and with different packaging conditions for 12 months of refrigeration storage.

		**Commercial Category (1)**			**Packaging (2)**		**SEM**	***p*** **Value**		
		***White***	***Red***	***Black***	**MAP**	**Vacuum**		**1**	**2**	**1 × 2**
**Brightness**	T0	5.0	5.1	5.1	4.9	5.2	0.101	0.290	0.656	0.194
	T8	5.2	5.5	5.8	5.3	5.8	0.128	0.123	0.042	0.637
	SEM	0.137	0.160	0.120	0.159	0.224				
	*p* value	0.455	0.147	0.190	0.163	0.039				
**Marbling**	T0	6.0 a	5.8 ab	5.1 b	5.9	5.3	0.144	0.039	0.051	0.431
	T8	6.4 a	5.4 b	5.2 b	5.6	5.7	0.135	0.000	0.746	0.000
	SEM	0.174	0.158	0.139	0.233	0.236				
	*p* value	0.246	0.209	0.209	0.035	0.047				
**Flavour**	T0	6.4	6.8	6.5	6.6	6.5	0.094	0.299	0.715	0.758
	T8	5.9	6.0	6.3	5.9	6.3	0.091	0.128	0.050	0.882
	SEM	0.117	0.109	0.094	0.180	0.163				
	*p* value	0.030	0.000	0.048	0.188	0.163				
**Hardness**	T0	5.5	5.8	5.8	5.8	5.7	0.122	0.529	0.692	0.272
	T8	6.4	5.9	5.8	5.9	6.2	0.120	0.080	0.118	0.037
	SEM	0.159	0.151	0.116	0.191	0.195				
	*p* value	0.004	0.692	0.692	0.958	0.195				
**Juiciness**	T0	5.5	5.9	5.9	5.7	5.8	0.123	0.233	0.613	0.414
	T8	5.6	5.6	5.8	5.5	5.9	0.119	0.679	0.081	0.140
	SEM	0.152	0.152	0.111	0.195	0.191				
	*p* value	0.597	0.273	0.273	0.360	0.191				
**Saltiness**	T0	5.4	5.8	5.5	5.6	5.6	0.086	0.132	0.969	0.523
	T8	6.0	6.1	5.7	6.1	5.7	0.091	0.097	0.036	0.462
	SEM	0.120	0.108	0.089	0.148	0.141				
	*p* value	0.012	0.133	0.048	0.318	0.141				
**Rancidness**	T0	2.1	2.0	1.9	2.1	2.0	0.138	0.880	0.729	0.410
	T8	2.7	2.7	2.7	2.8	2.5	0.138	0.988	0.282	0.358
	SEM	0.182	0.162	0.139	0.251	0.234				
	*p* value	0.099	0.041	0.010	0.014	0.234				
**Strange tastes**	T0	1.5	1.3	1.5	1.5	1.4	0.077	0.477	0.367	0.243
	T8	1.9	1.7	1.7	1.8	1.7	0.099	0.665	0.685	0.140
	SEM	0.128	0.092	0.084	0.196	0.108				
	*p* value	0.163	0.043	0.239	0.465	0.108				
**Persistency**	T0	6.0	6.0	5.9	6.0	5.9	0.097	0.899	0.887	0.158
	T8	6.5	6.4	6.4	6.4	6.5	0.094	0.968	0.510	0.975
	SEM	0.123	0.112	0.096	0.198	0.148				
	*p* value	0.021	0.048	0.043	0.136	0.148				

SEM, standard error of mean; *Black* (100% Iberian pigs finished in Montanera); *Red* (50% Iberian × Duroc pigs finished in Montanera); *White* (50% Iberian × Duroc pigs reared in a semi-intensive system with commercially feeding). Modified atmosphere packaging (MAP) (70% N_2_:30% CO_2_). Vacuum packaging. T0, initial; T8, 8 months of storage.

## Data Availability

Data supporting reported results will be available on request.
